# Inducing asymmetric gait in healthy walkers: a review

**DOI:** 10.3389/fresc.2025.1463382

**Published:** 2025-03-17

**Authors:** Gert Van Der Velde, Henri Laloyaux, Renaud Ronsse

**Affiliations:** ^1^ Faculty of Medicine, University of Ghent, Ghent, Belgium; ^2^ Louvain Bionics, Institute of Mechanics, Materials, and Civil Engineering, UCLouvain, Louvain-la-Neuve, Belgium; ^3^ Institute of Neuroscience, UCLouvain, Brussels, Belgium

**Keywords:** gait, asymmetry, adaptation, locomotion, perturbation

## Abstract

Gait symmetry between both legs is a typical hallmark of healthy walking. In contrast, several pathologies induce asymmetry in the gait pattern, regarding both spatial and temporal features. This can be due to either an asymmetrical change of the body morphology—e.g., after an amputation or an injury—or a damage in the brain—such as stroke or cerebral palsy. This deficit in gait symmetry usually induces higher metabolic effort in locomotion and might further accelerate severe comorbidities such as osteoarthritis and low back pain. Consequently, several assistive devices—such as active exoskeletons or prostheses—are currently developed to mitigate gait asymmetry and restore a healthier gait pattern. Typically, the development of such devices requires extensive tests and validations, and it is practically and ethically not always desirable to recruit disabled patients to run these tests in the preliminary stages of development. In this review paper, we collect and analyse the different reversible interventions described in the literature that can induce asymmetry in the gait pattern of healthy walkers. We perform a systematic literature research by exploring five databases, i.e., Pubmed, Embase, Web of Science, Google Scholar, and Scopus. This narrative review identifies more than 150 articles reporting 16 different interventional methods used to induce asymmetric gait pattern in healthy walkers or with the potential to do so. These interventions are categorized according to their mode of action, and their effects on spatiotemporal parameters, joint kinematics and kinetics are summarized adopting a macroscopic viewpoint. Interventions are compared in terms of efficacy, maturity of the results, and applicability. Recommendations are provided for guiding researchers in the field in using each of the identified manipulations in its most relevant research contexts.

## 1 Introduction

Walking is a key daily activity: an average healthy person takes between 4,000 and 18,000 steps a day (1). Several studies have therefore investigated the normal walking pattern, in order to unveil the mechanisms governing locomotion. Gait symmetry between both legs has often been considered a sign of healthy walking, as exemplified by the similarity between ground reaction force (GRF) profiles of both legs during walking (2, 3). Yet this statement has been challenged by other studies: while both legs behave in symmetry at the population level, much larger GRF asymmetries are found at an individual level (4). To explain gait asymmetries in healthy walkers, authors postulated that lower limb behavior during able-bodied locomotion is a reflection of natural functional differences between both lower extremities. These are potentially related to the contribution of each limb in carrying out the tasks of propulsion and balance during walking (5). The non-dominant lower limb contributes more to support, while the dominant one contributes more to forward propulsion (6). In sum, healthy walkers tend to display symmetric or moderately asymmetric gaits, the latter being due to functional differences between both legs.

Gait asymmetries are much more prominent in pathological populations, and this induces a series of secondary disorders. These pathologies often cause detrimental impairments regarding energy expenditure and balance (7), requiring up to twice the energy of a healthy gait (8, 9). This might have several sources, such as loss of strength and coordination of the pathological leg. In healthy walking, push-off with the first leg immediately begins before heel strike of the other leg, thus reducing the velocity of the collision with the ground. As a consequence, a decrease of push-off causes an increased contralateral collision and requires more energy compensation (7). Balance might also be affected by changes in the phase relationship between transverse pelvic and thoracic rotations (10, 11). These changes result in alterations of the trajectory of the center of gravity (12). Besides effects on energy cost and balance, asymmetric gait is also positively correlated with fall risk and dependency in activities of daily living in older adults (13).

Neurological disorders leading to altered gait pattern are stroke—resulting in hemiplegia—Parkinson disease, and cerebral palsy. Hemiplegia results in changes in almost all the parameters used to assess walking on both the pathological and non-pathological sides of the body (14). Hemiplegic patients show bigger flexion of the affected hip during mid-stance, smaller hip adduction during single support, and less knee flexion and dorsiflexion of the affected limb compared to baseline walking (15). Parkinson's patients have bigger variability of stride length (16) and alterations of the normal muscle functioning (17), yet generally there are no significant asymmetries found in their gait pattern. The asymmetries in the gait pattern of patients with cerebral palsy depend on the level of paralysis (18).

Scoliosis, arthrosis or arthritis and unilateral amputation are examples of non-neurological causes of altered gait. Scoliotic patients, depending on the severity of their condition, display an asymmetric trunk behavior eventually resulting in an asymmetric GRF pattern in several directions (19, 20), associated to an excessive energy cost of walking (21). Patients with unilateral hip arthritis display a longer total support time and shorter swing time for the unaffected limb (22). The step lengths are also asymmetrical with shorter steps for the affected limb. The same findings could be observed in unilateral transfemoral amputees who also stand a little longer on their intact leg compared to their prosthetic leg (23), causing asymmetry in both temporal (24) and spatial variables (25). Moreover, the level of asymmetry depends on the stump length: amputees with highly atrophied hip-stabilizing muscles walk with an extreme lateral bending of the trunk toward the prosthetic side (23).

Asymmetries can affect gait both in spatial features, such as a difference between right and left step length, and in temporal features, such as a difference in right and left single support duration. Therefore, it is important to quantify gait asymmetries using metrics associated with both the temporal and spatial aspects. According to (26), the most insightful parameters are step length, swing duration, and stance duration. The same authors established thresholds for what should be considered as pathological gait asymmetry, as compared to a healthy population.

Several technological solutions have already been provided to correct asymmetrical gait. These tools range from special orthopedic insoles correcting an anatomic leg length difference (27, 28), to high-end mechatronic prostheses (29, 30). Passive ankle-foot orthoses have been on the market for some time (31, 32), although research is still being carried out to improve their settings for specific disorders, like for patients with equinus (33). The most recent ankle-foot orthosis devices are no longer passive but have for instance an actively modulated impedance throughout the walking cycle. This results in improved walking symmetry for patients with a drop foot (34).

Typically, the development of such new locomotion supportive tools requires extensive tests and validations, and this is practically and ethically not always desirable to recruit disabled patients to run these tests in the preliminary stages of development. Although sensorimotor functions are fundamentally different between intact and disabled individuals, this early-stage research about assistive devices creates a need for inducing strong asymmetric gait in healthy walkers to support this early-stage research, and many people developed concurrent approaches to reach this goal. In this paper, we provide an overview of the methods that have been developed to induce asymmetric gait in healthy walkers. Literature search has been conducted to determine the effects of these interventions on spatiotemporal parameters, joint kinematics and kinetics (i.e., contact forces, muscle activations^1^ and resulting joint torques). We further compare these methods in terms of efficacy, maturity of the results, and applicability, and we provide recommendations for using each of them in specific research contexts.

## 2 Methods

### 2.1 Literature search

**Figure 1:**
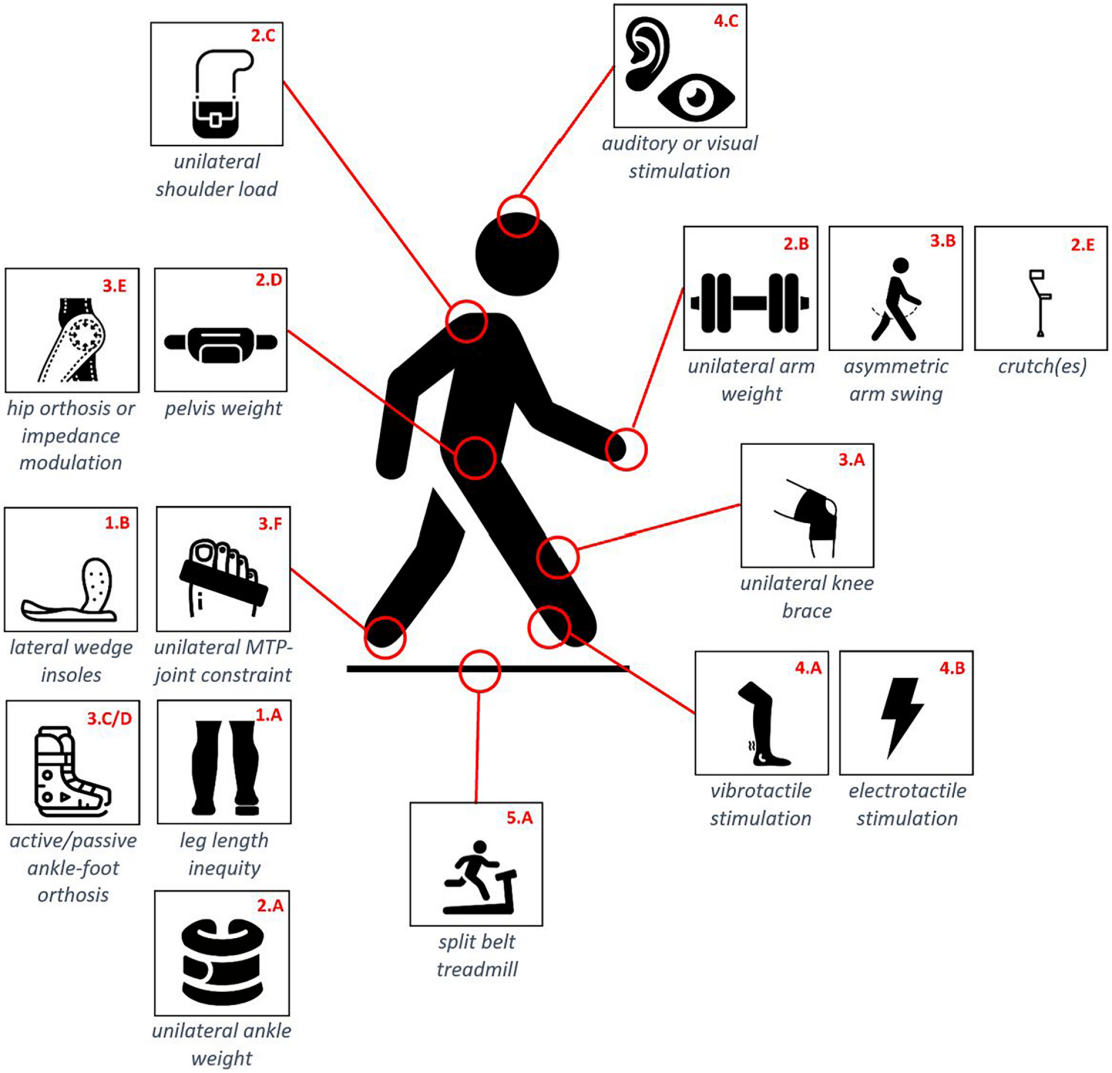
Schematic overview of the 16 identified methodological approaches. Each approach has a unique ID structured as X.y where X refers to the category each approach belongs to. These IDs are used throughout the whole paper.

We first performed a literature search in order to identify the methodological approaches that researchers used in order to induce asymmetric gait in able-bodied walkers. For this first step, we used Pubmed, using the advanced search feature with the following keywords: [(((able-bodied) OR (healthy)) AND ((asymmetrical) OR (asymmetry))) AND (gait)]. This led to a series of 692 research articles. A selection was done based on their title and abstract. Articles were kept for further investigations if the study population consisted of healthy participants who got an intervention to induce an asymmetric gait or describing an intervention that has potential to induce asymmetric gait. Gait asymmetry in this review was defined as an asymmetry in spatiotemporal parameters, joint kinematics, or kinetics. Articles were excluded when the study examined disabled subjects or if the research goal was to characterize healthy gait with no specific intervention to amplify asymmetry. This resulted in 24 articles mentioning 16 different methods that could induce gait asymmetry. These 16 methods are pictured in Figure 1, revealing that they can further be sorted into five parent categories, namely: (1) modifications of the participant's anatomy, (2) asymmetric loading of the participant's body, (3) modifications of the participant's joint impedance, (4) manipulations of the participant's sensory feedback, and (5) manipulations of the environment. The fourth category in particular gathers experimental interventions using vibrotactile, electrical, auditory, or visual stimulations. Vibrotactile tendon stimulation is indeed known to interfere with proprioception (35–37), generating similar responses in the muscle spindles as real movements.

**Figure 2:**
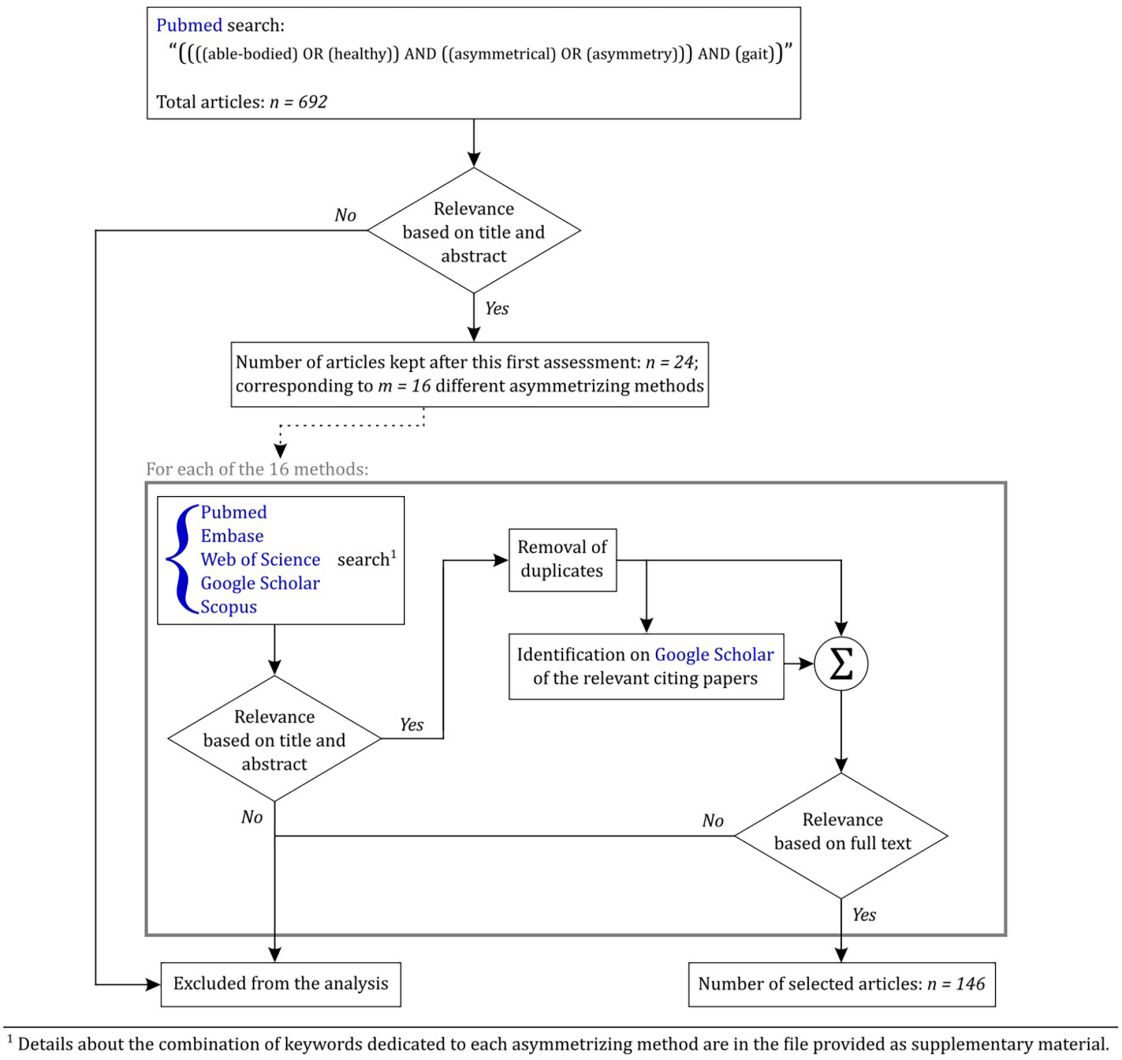
Workflow diagram illustrating our review process.

In a second stage, we conducted a separate literature search for each of these 16 methodological approaches, to specify how they influence gait with a specific focus on asymmetry. For this second step, we used five databases, i.e., Pubmed, Embase, Web of Science, Google Scholar, and Scopus, with articles with publication dates up to 2023 at the latest. Finally, we used Google Scholar to identify the papers citing those we kept after the second step, and the supplementary articles identified as being relevant for the present review were added in the database for further analyses. The detailed methods and results of this systematic search for each of the 5 parent categories and their corresponding 16 methods are reported in a Supplementary File. Our whole workflow process is represented in Figure 2.

### 2.2 Scoring strategy

Another contribution of the present paper is to provide recommendations about which manipulation should be used to induce gait asymmetry, as a function of the expected effects and objectives of the study. To achieve this goal, we based our approach on a reference method frequently used in the field of mechanical design (38). Subsequently, all the interventions reviewed in this paper were given a relative score based on three evaluation criteria, covering essential and complementary features of these interventions. To calculate these relative scores, each criterion was divided into up to 6 independent subobjectives, each of which being assessed separately, before computing a weighted sum to produce a global score on a single scale.

The first criterion quantifies the efficacy of the intervention. To score this criterion, the effects on spatiotemporal parameters, joint kinematics and kinetics were considered. Since assistive devices are mostly developed to get the gait pattern back to symmetrical regarding spatiotemporal parameters (39–43), the effect of an intervention on the spatiotemporal parameters was considered as the most important and was subdivided into spatial and temporal gait parameters. When no significant effect was found in the literature, a subobjective value of 0 was given. When the intervention caused a significant asymmetry in these parameters, but only in specific conditions—e.g., only if the intervention was applied on the dominant side but not on the non-dominant side—or there was no consensus amongst authors, a subobjective value of 1 was given. When the intervention caused a significant asymmetry regardless of the side on which it was applied and with consensus among the different publications, a subobjective value of 2 was given. These spatiotemporal parameters have the advantage of capturing the asymmetry with a single discrete value—even if it can be formulated according to different expressions (26)—which makes their analysis more robust than joint kinematics and kinetics (i.e., GRF, joint contact forces and joint torques) which require different elements of the entire kinematic/kinetic pattern to be taken into account. For this reason, we have decided to assign these parameters a subobjective value of 0, 0.5, or 1 for no effect, an effect in certain conditions only or without consensus, and a significant effect in most conditions with consensus, respectively. When no data was found in the literature, a subobjective value of 0 was given as well. Therefore, by summing up these subobjectives values, a single “efficacy” score is produced to capture the global effect of a given intervention on the different gait parameters.

Next, a “maturity” score was given, with the objective to capture both the effect regarding the size of the population on which a given intervention has been tested, and the number of studies that tested it. Indeed, the number of articles that investigated a given manipulation could be used as a proxy of how well studied the intervention is, and the number of subjects examined in the largest study, excluding reviews, could give an indication of the significance/quality of the reported results. Regarding the first item, if five articles or less were found for a specific intervention, a subobjective value of 1 was given; if six to ten articles were found, a subobjective value of 2 was given; and if more than ten articles were found, a subobjective value of 3 was given. Regarding the second item, if the largest study included up to 20 subjects, a subobjective value of 1 was given; and if the largest study included more than 20 subjects, a subobjective value of 2 was given. The third criterion was “applicability”, taking into consideration the ease of use. For this criterion, the set-up time and an approximated cost of the required equipment were considered. With the scoring of the set-up time, we aimed at evaluating and comparing the approximate time required to set up the experimental apparatus on the subject. If it was more than 30 min, a subobjective value of 1 was given, and if the estimated set-up time was less than 30 min, a subobjective value of 2 was given. Regarding cost, if the intervention required equipment costing more than 100€, a subobjective value of 1 was given; and otherwise a subobjective value of 2 was given. The estimated costs and set-up time did not take into account the materials and time needed for the actual data recording or the set-up of the measuring instruments needed to collect these data. Thresholds for both “maturity” and “applicability” subcriteria were set in order to reach a relative uniform distribution across the corresponding subobjectives values, with the exception of the set-up time which has been set on the basis of practical considerations and our own experience. However, it is still up to the reader to adapt these thresholds values in accordance with their own desiderata. This scoring methodology is summarized in Table 1.

**Table 1:** Overview of the scoring system for each of the three considered criteria.

Criterion	Subcriterion	Scoring	Clarification
Efficacy	Spatial gait parameters	0	The specific intervention does not induce proven asymmetry or the asymmetry on this subcriterion was not examined.
		1	The specific intervention causes proven asymmetry in specific research conditions.
		2	The specific intervention causes proven asymmetry in all research conditions.
	Temporal gait parameters	idem	idem
	Joint kinematics	0	The specific intervention does not induce proven asymmetry or the asymmetry on this subcriterion was not examined.
		0.5	The specific intervention causes proven asymmetry in specific research conditions.
		1	The specific intervention causes proven asymmetry in all research conditions.
	Ground reaction force	idem	idem
	Joint contact forces	idem	idem
	Joint torques	idem	idem
Maturity	Number of articles	1	Up to 5 articles were found in the databases, communicating on this specific intervention.
		2	Between 6 and 10 articles were found in the databases, communicating on this specific intervention.
		3	More than 10 articles were found in the databases, communicating on this specific intervention.
	Biggest study population	1	The biggest primary study on this specific intervention, found in literature, had a study population of up to 20 people.
		2	The biggest primary study on this specific intervention, found in literature, had a study population of more than 20 people.
Applicability	Set-up time	1	Estimated experiment set-up time for this specific intervention is greater than 30 min.
		2	Estimated experiment set-up time for this specific intervention is lower than 30 min.
	Costs	1	Estimated experiment costs for this specific intervention are more than 100 EUR.
		2	Estimated experiment costs for this specific intervention are less than 100 EUR.

For each criterion, score was then normalized by its respective maximum one. Then, a global score was computed by weighting the criteria of efficacy, maturity, and applicability by respectively 0.5, 0.3, and 0.2, capturing our own hierarchy between these criteria. This larger weight given to “efficacy” stems from being the main objective explored by this study. Next, the second most important weight is given to the “maturity” index capturing the level of dissemination of the different interventions in the literature. Finally, “applicability” was quantified as the least dominant criterion, as it is mostly a matter of authorized expenses and installation time. The rounding of each weight to the decimal point was then determined to reach a total value of 1.0. However, the reader is left free to assign different weights to these criteria, for instance to comply to particular research needs and/or working environment. In this case, the global score would be directly adjusted. In order to facilitate this potential adaptation, an editable and dynamic version of the comparative table is provided as Supplementary Material.

## 3 Results

As detailed in the Supplementary File, more than 140 papers were found after systematic literature searches. The main results reported in these papers are summarized hereafter.

### 3.1 Modifications of the participant's anatomy

#### 3.1.1 Leg length inequity (LLI, method 1.A)

Several authors examined the effect of LLI on the general gait parameters by quantifying gait asymmetry by means of various indices (44–46). Two studies examined the effects of an LLI of 4 mm. While gait initially became asymmetric, an adaptation phase resulted in the regain of symmetry after 10 min (44, 47). LLI's of 27 mm and 52 mm only led to a significant decrease in symmetry for the leg length difference of 52 mm according to the Combined Gait Asymmetry Metric (45); while a significant relative step lengths difference between both sides was found for a LLI of 32 mm in (46). The main findings are that the step length of the longer leg was increased compared to the step length of the shorter leg (46) and that the single support phase decreases on the side of the longer leg (47).

Concerning the kinematics of the lower limb joints, a LLI is typically compensated by walking with a more bent longer leg and a more extended shorter leg. Hip and knee of the longer leg are more flexed during both swing phase and stance phase compared to baseline walking while the ankle is more dorsiflexed. Moreover, the hip adduction and external rotation decreased with bigger LLI's. Additionally, the ankle reaches the peak dorsiflexion in stance phase relatively later compared to baseline walking. On the short side, the main observations during stance are an increase in knee extension, hip extension and abduction and ankle plantarflexion; while in swing phase only decreased ankle dorsiflexion and increased hip abduction are observed (48–51).

The magnitude of the LLI is significantly correlated to changes of the pelvic position, resulting in postural changes in the spine (52). The main observations are an increase in the maximal lateral bending angle and bending speed of the thoracal and lumbar spine with increasing lateral deviation towards the longer leg when the LLI is bigger and an increase of the surface rotation of the spine (here orientated towards the longer leg) (52–55).

Although (50) suggested that significant kinematic changes of the longer limb were already observed in certain gait phases for a LLI of 5 mm and (55) only found significant results for a LLI of 40 mm, a minimal LLI of 20 mm is cited in (52, 56). Note that this finding might be partly due to the fact that these papers did not systematically use the same kinematic metrics and/or age groups.

Based on inverse dynamics, contact forces in the leg joints for several LLI's between 5 and 40 mm were computed and analyzed in (57). They found that the mean contact forces in the ankle, knee and hip are significantly larger in the shortest leg compared to the longest one. Contact forces in the joints of the shortest leg increase with bigger LLI's, again with a threshold below which no difference is found with normal walking (57, 58). On the contrary, contact forces in the medial compartment of the tibiofemoral joint show a declining trend with increasing LLI and contact forces in the lateral compartment of the tibiofemoral joint show a stable trend with increasing LLI (59). The contact forces in the hip joint display an increasing trend with increasing LLI's except for a LLI of 30–35 mm, due to a pelvic tilt that moves the COM to the contralateral side during the stance phase (59). Two studies found a significant asymmetry in ground reaction force with a trend of increasing GRF for the short leg and decreasing GRF for the long leg with increasing LLI (60–62).

#### 3.1.2 Lateral wedge insoles (method 1.B)

A customized printed single-sided lateral wedge insole with 6° inclination has been used to perturb gait (63). Asymmetries appeared immediately after use of the insole and remained after 20 min, yet not at the level of step length or speed. Furthermore, the vertical GRF was significantly larger for the instrumented side and the lateral moment of the center of pressure significantly increased, shifting to the contralateral side, compared to walking without the customized insole. The adduction knee torque at the side with insole is also significantly lower than for walking without this insole.

### 3.2 Asymmetric loading of the participant's body

#### 3.2.1 Change in body morphology—unilateral ankle weight (method 2.A)

Thirteen authors examined the effect of a unilateral ankle weight (UAW) on the general gait parameters by quantifying gait asymmetry by means of various indices. All examined weights were found to induce an asymmetric gait pattern in the participants, even for a weight that is as low as 1% of the participants’ bodyweight (64–67). Moreover, all studies reported that the asymmetry was bigger for heavier weights. The effects of a UAW are also influenced by the age of the participant (64, 68). Overall, the addition of a UAW causes a change of intralimb coordination with deviations from the baseline antiphase pattern, with the loaded leg lagging behind the other (69, 70).

Adding a UAW results in an asymmetrical step length that can take up to 10 min to become noticeable (65, 71, 72). Curiously, adding weight resulted in contradictory results regarding step length: (65) reported the weighted limb to exhibit shorter steps, while (64) reported longer steps. Possible explanations are (i) that this was measured at different time intervals after adding the weight, (i.e., during early vs. late adaptation), (ii) that participants were allowed to adapt their gait speed in every condition only in (64), and (iii) that the added weight was not similar between studies (i.e., 5% and 7.5% of the body weight). Single limb support time becomes asymmetrical once the weight is added (71, 72) and the double support time decreases significantly (73). This is due to an increase in swing time for the loaded limb and an increase in stance time for the unloaded limb (64, 72).

Adding a UAW has several effects on joint kinematics. Compared to unweighted walking, an increased hip extension and decreased hip and knee flexion can be observed for both the affected and unaffected limb (74). The decreased flexion of knee and hip is compensated by increasing ankle dorsiflexion during mid-swing to aid in toe clearance (73, 74). Absolute angular impulses of the loaded limb hip and knee increases significantly after load addition (75). Net joint moments at the knee and hip continue to change beyond initial exposure to the load, reaching steady-state within five minutes (60). The effects on joint kinematics depend also on the participant's age: ankle angle at touchdown is greater with UAW for younger adults than for older adults (68).

Finally, (76) developed a cable-driven loading of the ankle that can be applied during the swing phase only. This perturbation resisted forward movement of the swing leg under either an abrupt or a gradual loading. They reported a greater asymmetry in the abrupt condition than in the gradual condition regarding swing phase duration, step length, and muscular adaptation.

#### 3.2.2 Change in body morphology—unilateral arm weight (method 2.B)

Eleven studies examined the effect of a unilateral arm weight on both spatiotemporal, kinematics and kinetics parameters, and these led to contradicting results. A reduction in step length for the contralateral leg was seen only for the bigger weights (77) but differentiation in step duration can already be found whilst carrying a mass equivalent to that of a smartphone (78). In contrast, carrying a load of 1.5 kg in one hand did not negatively affect postural stability and gait variables (79), and no asymmetry in arm swing amplitude with a unilateral arm weight of 0.45 kg was found in (80). Mixed results were reported in (81): no significant differences in step length, step time and double support time, while—only for the highest loads—stance time for the loaded leg, and single support and swing time for both legs significantly decreased. This increase in double support time was not confirmed in (82). Finally, (83) reported an increase in compression area of the feet for increasing loads.

Several kinematic changes were observed when adding a unilateral arm weight, such as an increase in foot angle of the ipsilateral leg (77, 81) and an increased trunk flexion towards the contralateral side (84, 85). A significant pelvic tilt for loads of 15% of body weight with pelvic rotation decreasing with increasing load has also been observed in (86). At ipsilateral trunk bending, a statistically significant increase in the maximum values of contralateral hip adduction and contralateral shoulder abduction, and a decrease in ipsilateral hip adduction were reported as loads increased (81). These kinematic alterations cause consistent asymmetries in the body COM oscillations (82).

Only one study examined the effects of a unilateral arm weight on the GRF and reported a higher level of asymmetry in medial/lateral GRF and free vertical moment (i.e., the GRF-induced moment around the body vertical axis) in this condition (85). Another single study reported the effects on joint torques, i.e., an increase of the contralateral hip abduction torque and a decrease of the ipsilateral hip torque (84). Finally, alterations in general gait parameters for a trolley with a load of 20% of body weight were reported in (87). Pulling a trolley can be considered as a unilateral arm weight both pulling downwards as backwards. However, no gait asymmetry was observed.

#### 3.2.3 Change in body morphology—unilateral shoulder load (method 2.C)

Unilateral shoulder load causes alterations in spatiotemporal parameters, gait stability and regularity depending on the heaviness of the load (88, 89). However, carrying a bag on one shoulder, compared to carrying it on the forearm or by hand, has the smallest effect on gait parameters (78). Yet coordination—especially couplings between both thighs, between the thigh and the shank of the loaded side, and between the shank and the foot of this loaded side—is altered and asymmetric load carriage leads to an asymmetry in step length with the right foot taking longer steps when carrying the asymmetric load over the right shoulder (90, 91).

A decrease for peak ankle dorsiflexion, mean knee varum angle, peak value of hip extension and range of pelvic rotation can be observed on the loaded side (92, 93). In addition, an increase for knee flexion at initial contact, hip adduction angle, mean pelvic anterior tilt and mean pelvic obliquity on the loaded side can be observed. Some of these effects are compensated on the unloaded side, with increased contralateral hip and knee moments (93). Like for the unilateral arm weight, (86) also reported a significant pelvic tilt for loads of 15% of body weight while pelvic rotation decreased with increasing load. Yet, the variability of the COM in vertical direction was significantly reduced when carrying a load (94).

Only one study examined the effects on GRF but there was no significant difference found in peak vertical GRF between baseline walking and walking with a unilateral shoulder weight of 7 kg, which was around 15% of body weight for most of the subjects (95).

#### 3.2.4 Change in body morphology—added weight to the pelvis (method 2.D)

Two studies examined the effect of a cable-driven active tethered pelvic assist device, used to apply an external force on the pelvis (96, 97). The adaptation to the applied force resulted in asymmetry in stance phase timing. Although no asymmetry in step width was reported, stability was kept by increasing the circular propulsion of one leg, depending on the direction of the force (97). Asymmetric changes were observed in the anteroposterior and vertical pelvic motion and larger hip flexion-extension range of motion (ROM) was found at the side of external force (96). Subjects demonstrated asymmetric lateral ground reaction force to compensate for the lateral forces applied on the pelvis (97). The lateral GRF increased and plateaued but the increment of vertical GRF (compared to baseline walking) decreased over time.

A similar device has been developed to provide braking forces to the user's pelvis during the stance phase of one leg (98). One healthy participant displayed walking asymmetry, in particular through a larger propulsive GRF in the resisted leg.

#### 3.2.5 Crutches (method 2.E)

Elbow/axillary crutches—either a unilateral one or a pair supporting the same leg—cause a shift in the COM which results in a reduction of step length for both legs and overall gait velocity (99–102). The effects on stance phase and swing phase depend on the experiment conditions. When walking with two crutches and simulating an injured leg, putting less weight on that side, stance phase decreased and swing phase increased whilst stance phase increased in crutch assisted gait without simulating any pathology (100, 102). There is no consensus regarding step width (99–101).

When a unilateral crutch is carried in the dominant hand, the gait changes more (99). Yet, subjects had a sufficient level of coordination to perform the required unloading of weight without pronounced modification between the steps of the dominant and non-dominant sides (99, 101). If participants are asked to simulate an injured leg and put a fraction of their body weight on two crutches instead of the foot of that injured leg, the single support phase for both sides were found significantly different, i.e., shorter for the involved side (100). When examining two-point and four-point crutch gait in healthy subjects without simulation of any pathology, no gait cycle asymmetries could be observed (102).

Slightly greater hip abduction and external rotation on the involved side and slightly less hip adduction and internal rotation on the non-involved side can be observed while walking with elbow crutches simulating a leg injury (100). Furthermore, for both sides the ROM patterns changed for the pelvis sagittal tilt as well as the hip, knee, ankle, and foot joints. The results of these kinematic changes appeared to be linked to a shift of the COM from the involved side toward the non-involved side (100).

Due to the support provided by crutches, the vertical GRF on the involved side—bearing less weight—is lower during almost the whole stance phase and exhibits a more plateaued pattern (100, 103). While differences of GRF in the mediolateral plane were negligible, anteroposterior GRF displayed a more pronounced asymmetry but mainly in amplitude, not regarding the pattern shape (100, 103). No asymmetry in foot pressure patterns has been found in (102).

### 3.3 Modifications of the participant's joint impedance

#### 3.3.1 Knee brace (method 3.A)

The effect of a unilateral knee brace on spatiotemporal parameters was not found in the literature. In contrast, several studies reported the effect on joint kinematics. A brace with 30°-extension constraint induces increased peak knee flexion and decreased peak knee extension for the braced limb (104). During swing phase, peak dorsiflexion of the ankle was greater and the ROM at the hip joint was also bigger for the braced condition, compared to baseline walking (104, 105). With a similar setting, (106) found smaller peak flexion for the braced knee while peak flexion occurred earlier in the swing phase compared to baseline walking. They also reported a lower peak extension for the unbraced knee during the early stance phase compared to baseline walking. Other authors developed a similar setup to test the effect of unilateral robotic walking assistance with healthy walkers (40). They also found smaller peak flexion for the braced knee as compared to the control condition.

The anterior reach was reduced for both legs when the brace was constrained but restriction of overall mobility of the leg was limited (107). Bilateral net knee extension moments gradually increased as the angle of contracture increased (108). The net knee extension moments in the non-constrained limb were significantly larger such that a knee flexion contracture greater than 15° led to mechanical overloads in both limbs. The knee shearing forces in contracture side and the knee compressive force in non-contracture side also significantly increased (108).

Only one study examined alterations of GRF and reported that vertical GRF values increase bilaterally at mid-stance but decrease bilaterally during propulsion (107). Furthermore, (105) reported a significant increase of ipsilateral peak hip power and the contralateral hip showed significant increases in generating mechanical work in early stance phase, with bigger effect is fixing the knee in a more flexed position. Finally, (109) shed light on individualized compensatory strategies adopted by two healthy walkers constrained by a fully locked knee.

#### 3.3.2 Asymmetric arm swing (method 3.B)

Arm swing amplitude as well as arm swing asymmetry vary considerably in healthy populations, e.g., as a function of age (110–112). Not all arm swing asymmetries are pathological: only asymmetries where one side has twice the amplitude of the other should be considered abnormal (110). A reduced lower limb coordination can be seen when walking with an induced asymmetrical arm swing (113). Unilateral casted participants took significantly smaller steps with the leg on the casted side, and single leg support time was also smaller for that side (114). The smallest changes were noted with the arm in a cast below the elbow and no sling, and the greatest changes were noted with the arm in a cast above the elbow and in a sling. Moreover, (115) reported that single leg stance and double support times were decreased and increased, respectively, compared to baseline walking.

Similarly, (116) studied the effect of a shoulder immobilization on gait. They found that spatiotemporal parameters such as velocity, step length, and stride length were significantly decreased after immobilization. However, they found no significant result regarding changes in gait symmetry.

#### 3.3.3 Active ankle-foot orthosis (AFO, method 3.C)

A unilateral powered AFO has been used by (117, 118) to induce gait asymmetry. They reported that the stance phase was significantly reduced for the contralateral leg. Results for the ipsilateral leg were not significant as compared to the control condition. The subjects increase step length for both legs during the assisted walking although this was not significant.

If the AFO was used to increase joint stiffness, this resulted in reduced peak ankle plantarflexion and dorsiflexion, reduced total ROM, and increased dorsiflexion at initial contact (118). Moreover, flexion of the knee increased at initial contact, while its peak extension decreased, and its peak flexion increased during stance when ankle stiffness was increased. Stiffness did not affect hip kinetics and there was low evidence for its effects on hip or pelvis kinematics and ankle and knee kinetics. More knee flexion was observed in both swing and stance phases when walking with a powered knee-ankle-foot orthosis compared to a conventional one (119).

Regarding GRF, (120) reported only one result with a 1-degree of freedom active AFO: COM trajectories were generally more localized in the lateral direction than with a 2-degrees of freedom one.

#### 3.3.4 Passive ankle-foot orthosis (method 3.D)

The main effects of a unilateral passive AFO on spatiotemporal parameters were a decrease in step length, step time and single support phase of the contralateral leg (121), over a large range of stiffnesses (118, 121, 122). The stance phase duration of the instrumented leg has been found to be relatively shorter (122). Walking speed decreases when walking with a passive AFO (123, 124) although this was not significant in all studies (125).

Just like with an active AFO, larger stiffnesses of the device generally resulted in reduced peak ankle plantarflexion and dorsiflexion, reduced total ROM and increased dorsiflexion at initial contact, larger flexion of the knee increased at initial contact, smaller peak knee extension, and larger peak knee flexion during stance (118, 124, 126). Unilateral restricted ankle motion influenced kinematics mainly in the swing phase (125). Hip and knee peak flexion were increased on the instrumented side and occurred earlier in the swing phase compared to baseline walking. Stiffness did not affect hip kinetics and there was low evidence for its effects on hip or pelvis kinematics and ankle and knee kinetics (118).

The forward tilting angle of the trunk at the time of toe-off of the instrumented side was significantly larger than the contralateral one (123). In contrast, (126) reported the biggest changes in the opposite leg suggesting that all its joints might undergo changes. The maximum dorsiflexion angle of the ankle was significantly smaller in the leg with the ankle-foot orthosis than in the leg without the device, such as the maximum plantar flexion angle in the early stance phase and at the time of toe-off (123). In addition, (127) reported that peak plantarflexion angular velocity and eversion ROM was smaller when walking with a unilateral dynamic orthosis compared to a standard AFO and baseline walking.

In one study, the peak vertical ground reaction force at heel strike was found to be significantly larger in the leg without the AFO than in the instrumented one (123). Yet, no significant difference was found in the anteroposterior GRF impulses compared to baseline walking (128); and this pattern of force distribution under the foot directly depended on the angle of the AFO during the stance phase (129). This further increased the foot pressure of the lateral foot sole, such as the peak foot pressure of the heel on the instrumented side. The effects of AFO stiffness on GRF were limited (118).

Using a similar device, (128) reported a greater range of angular momentum in both the frontal and sagittal planes, which were correlated with the reduced peak hip abduction and reduced ankle plantarflexion moments, respectively. The peak knee extension torque was also reported to be larger (126). Regarding muscle activation patterns, (126) found that an AFO significantly reduced lower leg muscle activity whilst (130) reported that peak quadriceps muscle force increased when the orthosis had a strong plantar flexion resistance.

#### 3.3.5 Hip orthosis or impedance modulation (method 3.E)

Wearable active hip orthoses are mainly used for locomotion assistance with diverse populations of patients (131–133). Nevertheless, a couple of studies also investigated how these tools could be used to induce asymmetric walking, typically by artificially modifying one hip impedance via closed-loop control. In (134), authors tested whether unilateral hip perturbations elicit neural adaptation in healthy participants. They found that applying a virtual stiffness parallel to the hip joint elicited time-dependent and asymmetrical changes in hip range of motion and step length, indicating an adaptation response. This research paves the way towards applying mechanical impedance asymmetrically to the joints for gait training and rehabilitation. Recently, the same group extended the protocol to bilateral and asymmetrical stiffness modulation (135), highlighting aftereffects in step length and propulsive/braking ground reaction forces. This was reported to be a signature of neural adaptation.

A couple of other papers were found about modifying the dynamic environment of the hip through another manipulation than via an active orthotic device. In (136), a passive device—namely a custom-made hip-thigh compression sleeve—was used to restrict one hip range of movement of healthy walkers. Results showed that this naturally result in asymmetric walking. However, since the purpose of that paper was to validate an assistive method through a dedicated device, no detailed results have been reported about the asymmetric pattern. Similarly, (137) reported investigations about using an active exoskeleton to restore walking symmetry. In that case, they artificially induced gait asymmetry in healthy walkers by attaching one of their thighs to the treadmill structure via an elastic rope. They showed that this manipulation induces gait features similar to the one of stroke survivors, namely reduced and earlier peak position, and smaller range of motion of the hip. They did not report other gait metrics.

#### 3.3.6 Unilateral metatarsophalangeal (MTP) joint constraint (method 3.F)

Specific research has been conducted on the effects on general gait parameters when immobilizing the MTP joints in one foot. In (138), results highlighted that the contralateral step length was significantly decreased and an asymmetrical COM pattern was also observed. The double leg support phase increased significantly but the single leg support phase of the ipsilateral leg decreased.

When walking with an insole restricting dorsiflexion in MTP joints, the ankle was more dorsiflexed during late mid-stance and less plantarflexed during propulsion, the knee was more flexed during mid-stance, and the hip was less extended during late mid-stance (139). Maximum hip and knee flexion angles in the swing phase of both limbs were increased (138, 140).

Only one study examined the effects on GRF and reported that the second peak in vertical GRF typically observed during the stance phase is larger compared to baseline walking (140). Increased stiffness resulted significantly in greater peak ankle moment, greater ankle push-off work, greater peak ankle foot power, greater ankle foot push-off work and decreased positive work by the ankle joint during the late stance phase (138, 141).

### 3.4 Manipulations of the participant's sensory feedback

#### 3.4.1 Vibrotactile stimulation (method 4.A)

Eight authors examined different types and locations of vibration and reported the effects on general gait parameters. The effects of continuously and intermittent vibration on the posterior neck muscles and triceps surae tendon of the non-dominant leg were investigated in (142). They reported no effect of neck muscle vibration on balance, a more backward body position due to continuous and intermittent vibration of the triceps surae and no change in spatiotemporal gait parameters. Similarly, (143) did not show an effect on temporal parameters. Bilateral mastoid vibration significantly increased the amount of sway variability and decreased the temporal structure of sway variability only in the anterior-posterior direction (144). Stimulating the mastoid can also induce changes in the margins of stability during walking, asymmetrically if the vibration is unilateral (145). With respect to interlimb coordination, only vibration of the biceps femoris showed a significant increase in phase lead of the vibrated limb (146). Vibration of the triceps surae tendon induced significant, though minor, changes in duration and length of stance and swing phase (147); and reduced the center of pressure velocity and displacement especially with eyes closed in healthy elderly subjects (148).

Vibration of the quadriceps femoris at the knee decreased knee flexion at the-mid swing phase, vibration of the tibialis anterior decreased plantar flexion at the toe-off phase, and vibration at the triceps surea decreased dorsiflexion during the swing phase (143, 146). Vibration on the stimulated leg did not induce any effect on joint displacement of the non-stimulated leg and thus induced gait asymmetry, leading to the conclusion that acute effects of vibration during gait involving healthy participants are varied (149).

#### 3.4.2 Electrical stimulation (method 4.B)

According to our searches, no study has been conducted yet using electrical stimulation with the aim to induce asymmetric gait in healthy subjects. Moreover, comparing results is difficult since applying this type of stimulation is done at several locations and with different intensities, during different gait phases. Thus, this mode of action can either interfere with proprioception, direct muscle activation, or cause a pain reaction.

The effects of unilateral electrical stimulation of the plantar intrinsic foot muscles from mid-stance to pre-swing has been examined in (150). They reported a significantly bigger decrease in the second peak of the vertical ground reaction force compared to baseline walking but no change in gait velocity, stance phase duration, minimum navicular height, and GRF in other directions. The effects of unilateral electrical stimulation of the tibialis anterior nerve on plantar pressure and gastrocnemius medialis activity led to a decrease in peak plantar pressure but no changes of gait parameters (151). Unilateral functional electrical stimulation to the gluteus medius muscle resulted in a decrease of the medial knee joint reaction force, a decrease in peak pelvic drop in the frontal plane towards the swing leg, during stance phase, and a reduction of the mediolateral component of the GRF (152). Finally, a unilateral phase-specific nociceptive electrical stimulation at the ankle led (153) to conclude that—during the first couple of stimuli—the peak heel contact decreased on that side and increased on the non-instrumented side while heel contact duration was bilaterally reduced. One minute after the start of these stimulations, only a decrease in peak heel contact pressure on the stimulated side persisted.

#### 3.4.3 Auditory or visual stimulation (method 4.C)

Providing a rhythmic auditory stimulation to healthy walkers induced bilateral changes of both spatial and temporal parameters and joint kinematics, but neither walking asymmetry (154–159) nor change in gait variability (160). The aim of (161) was to induce asymmetry in the gait pattern of healthy subjects using a rhythmic auditory stimulation. Although subjects synchronized their gait within a few steps when the auditory cues were delivered, results suggest that it is not possible to desynchronize the gait of healthy subjects using such a rhythmic auditory stimulation.

In contrast to rhythmic auditory stimulation, phase-shifted auditory cue significantly changes gait symmetry and trunk displacement (162). The more the auditory cue was out of phase, the larger the observed trunk displacement. Moreover, step length, step time and swing phase time symmetry, also gradually increased with increasing phase delay and gradually decreased with increasing phase advance. On the other hand, single support time and stance phase time symmetry showed contrasting characteristics compared to above parameters. Similarly, asymmetric walking can be induced by different levels of loudness provided to both ears (163). In particular, these authors found a significantly increased stance time on the side with reduced volume. Another study focused on walking perturbations induced by dichotic listening, i.e., attention directed to right or left ear (164). They used Principal Component Analysis and showed asymmetrical disruptions on the components structure, capturing asymmetric modulations in step/stride width and length. Finally, (165) examined the effects of a fractal metronome on gait in healthy walkers and reported that participants did synchronize with the metronome despite its fractal randomness, causing asymmetries in general gait parameters.

We found only one study that specifically investigated the use of visual feedback to induce asymmetric walking (166). Visual targets were projected on a treadmill to instruct healthy walkers to take shorter or longer step than preferred. The authors found that participants naturally adopted asymmetric walking regarding both step length and duration, in order to minimize their metabolic cost. Contact force profiles were also found to become asymmetric in specific experimental conditions.

### 3.5 Manipulations of the environment—split-belt treadmill (method 5.A)

Literature relying on split-belt treadmill protocol is abundant since it has been largely used as a proxy to study adaptation and learning in locomotion tasks, including with patient populations (167, 168). A general factor influencing gait asymmetry is the subject's perception of belt speed difference, as reported in (169, 170). A threshold of 0.88 for belt speed difference ratio is highlighted to induce gait asymmetry (170).

Two studies showed that for both younger adults and elderly, adaptation to split-belt treadmill walking was done by re-establishing symmetry in step length and double support time (171, 172). Elderly mainly increased swing speed of the fast leg whereas younger adults mainly increased the swing time of the fast leg. However, this asymmetry occurring at early adaptation tended to disappear during late adaptation (171–174), together with an increase in whole body angular momentum (175). While swing time of the fast leg and swing speed of the slow leg slightly decreased during late adaptation, swing speed of the fast leg continued to increase (172). Leg excursion asymmetry, which was biggest in the elderly, normalized in late adaptation as well (171–173). Similar results were found with visual occlusion (176). Thus, at initial contact with the split-belt treadmill, subjects showed a step length asymmetry and a double support asymmetry which normalized in the first couple of minutes except regarding the swing duration of the fast leg.

With greater speed differences, participants adopted increasing values of step time asymmetry while the step lengths and stride time remained constant and nearly symmetric (177, 178). Similarly, (179) noted a prolonged stance phase and a shortened swing phase of the slower limb and inversely for the faster limb. Stride times for both legs shortened during adaptation: where the fast leg had a shorter double limb support time at beginning, this became equal for both legs, although stance phase and swing times did not normalize. With greater speed differences, step time asymmetry increased while step length asymmetry remained constant. Stance phase and swing times did not normalize after adaptation.

Five authors examined the effect of split-belt treadmill speed differences on the joints of the lower limbs. One of the articles reported that knee joint contact forces were symmetric during the entire adaptation (173). Similarly, (180) found a significant difference in the intrasubject variability of knee flexion at heel strike; and (174) found that the symmetry index of the anterior force became asymmetric in the first minute and moderately changed, lasting for the remaining time of the adaptation period. The mediolateral GRF and hip moment impulse of the fast limb increased over time with adaptation, but (181) did not find differences in any joint moments or mediolateral GRF during early or late adaptation compared to baseline walking. Alterations in the GRF differed between the anterior and the posterior components, both during and after the split-belt exposure (182).

Six studies also examined the effects of adding a cognitive task during split-belt walking on the adaptation process. Regardless of cognitive task placement or duration (intermittent or continuous), subjects tended to prioritize gait symmetry over cognitive performance (183). A second study also reported no additional effect of texting on a smartphone on gait parameters, while walking on a split-belt treadmill (184). In (185), split-belt treadmill was combined with visual cues to promote adaptation of step timing or position. The main finding was that such visual feedback affects deadaptation but not adaptation to the split-belt configuration. Dual-tasking while walking on a split-belt treadmill only caused an additional effect on limb excursion asymmetry compared to normal split-belt treadmill walking, during early post adaptation (169); while the presence of a dual-task during adaptation slowed the rate of adaptation to the split-belts and was characterized by greater variability compared to the single-task group (186). Finally, (179) reported a main effect of dual tasking to double support proportion variability. Furthermore, stance phase significantly increased during dual tasking for the limb on the faster belt, while it decreased for the limb on the slower belt, although the latter effect was smaller. There were also significant interactions between dual task and belt speed.

### 3.6 Combinations

We found three studies that specifically combined at least two of the experimental conditions described earlier. In (187), the effect of a unilateral ankle weight combined with an induced leg length inequity on the ipsi- or contralateral leg was studied. Although asymmetry in gait was reported in several parameters for unilateral ankle weight and LLI alone, authors did not report significant interactions between the amount of mass and leg length added. Similarly, (45) only reported a significant asymmetry compared to baseline walking for a LLI of 52 mm and a UAW of 2.3 kg. Combinations of a smaller LLI or bigger UAW did not result in significant increase in asymmetry. Finally, (188) studied five walking conditions combining three types of perturbations: extra ankle weight (0.9 or 1.2 kg), adding a 25.4 mm LLI, and with a walking boot locking one ankle. They reported similar asymmetries as in the corresponding studies focusing on a single perturbation type. The focus of their paper was on the consequences of these artificially-induced gait asymmetries on lower back pain. They found that lower back kinetic demands associated with asymmetrical gait were similar to, or only moderately different from, normal walking for most conditions despite the induced asymmetries.

### 3.7 Scoring

Each intervention was scored according to the criteria explained in the methodology. This scoring system results in a gradation according to the efficacy of the method, its level of maturity, and its practical applicability. These scores are gathered in Table 2.

**Table 2:** Overview of the given scores per intervention and the global score.

Intervention	Efficacy (weighting factor 0.5)							Maturity (weighting factor 0.3)			Applicability (weighting factor 0.2)			Global score
	Spatial gait parameters	Temporal gait parameters	Joint kinematics	Ground reaction force	Joint contact forces	Joint torques	Overall relative score	Number of articles	Biggest study population	Overall relative score	Set-up time	Costs	Overall relative score	
1.A Leg length inequity	2	2	1	1	1	0^a^	*0*.*88*	3	2	*1*.*00*	2	2	*1*.*00*	***0***.***938***
1.B Lateral wedge insoles	0	0	0^a^	1	0^a^	1	*0*.*25*	1	2	*0*.*60*	2	2	*1*.*00*	***0***.***505***
2.A Unilateral ankle weight	1	2	1	0^a^	0^a^	0^a^	*0*.*50*	3	2	*1*.*00*	2	2	*1*.*00*	***0***.***750***
2.B Unilateral arm weight	1	1	1	1	0^a^	1	*0*.*63*	2	2	*0*.*80*	2	2	*1*.*00*	***0***.***753***
2.C Unilateral shoulder load	2	2	1	0	0^a^	0^a^	*0*.*63*	2	2	*0*.*80*	2	2	*1*.*00*	***0***.***753***
2.D Added weight to the pelvis	0	2	1	1	0^a^	0^a^	*0*.*50*	1	1	*0*.*40*	2	1	*0*.*75*	***0***.***520***
2.E Crutch(es)	2	2	1	0	0^a^	0^a^	*0*.*63*	1	1	*0*.*40*	2	2	*1*.*00*	***0***.***633***
3.A Knee brace	0	0	1	1	0^a^	1	*0*.*38*	2	2	*0*.*80*	2	2	*1*.*00*	***0***.***628***
3.B Asymmetrical arm swing	1	1	0^a^	0^a^	0^a^	0^a^	*0*.*25*	1	2	*0*.*60*	2	2	*1*.*00*	***0***.***505***
3.C Active ankle-foot orthosis	0	1	1	0	0^a^	0^a^	*0*.*25*	1	1	*0*.*40*	1	1	*0*.*50*	***0***.***345***
3.D Passive ankle-foot orthosis	2	2	1	0.5	0^a^	1	*0*.*81*	3	1	*0*.*80*	2	1	*0*.*75*	***0***.***796***
3.E Hip orthosis or impedance modulation	2	0^a^	1	1	0^a^	0^a^	*0*.*50*	1	1	*0*.*40*	1	1	*0*.*50*	***0***.***470***
3.F Unilateral metatarsophalangeal joint constraint	2	2	1	1	0^a^	1	*0*.*88*	1	1	*0*.*40*	2	1	*0*.*75*	***0***.***708***
4.A Vibrotactile stimulation	1	1	1	0^a^	0^a^	0^a^	*0*.*38*	2	1	*0*.*60*	1	1	*0*.*50*	***0***.***468***
4.B Electrical stimulation	0^a^	0^a^	1	1	1	0^a^	*0*.*38*	1	1	*0*.*40*	1	1	*0*.*50*	***0***.***408***
4.C Auditory or visual stimulation	2	2	0^a^	0^a^	0^a^	0^a^	*0*.*50*	2	2	*0*.*80*	2	2	*1*.*00*	***0***.***690***
5.A Split-belt treadmill	2	2	0^a^	0.5	0	0.5	*0*.*63*	3	2	*1*.*00*	2	1	*0*.*75*	***0***.***763***

Italic values report intermediate scores, i.e., the overvall relative score of each scoring criterion, while bold values correspond to the global weighted score.

^a^ Not reported in the literature.

## 4 Discussion

Walking in a straight line on a flat surface or a treadmill is performed with a quasi-symmetric pattern. As overviewed above, several interventions have been investigated to break down this symmetry and induce asymmetric gait in healthy walkers. These experimental investigations are often used to test rehabilitation or assistive strategies aiming at restoring walking symmetry before recruiting actual patients. In general, these interventions cause alteration on different aspects of the gait pattern, and—as a reaction—behavioral adaptation for compensating the new body and/or environment dynamics. Such interventions therefore modify the gait both through their primary means of action, and indirectly. For instance, a lateral wedge insole mainly alters the spatial orientation of the leg joints, but inevitably also creates a leg length inequity to some extent. Similarly, adding a unilateral arm weight would also alter the arm swing symmetry. Using a cast on the elbow will cause effects due to an added weight as to an induced arm swing asymmetry. The same accounts for a unilateral bag, a weight or even a crutch since this added weight will alter the natural swing movement of the arm on that side. A unilateral knee brace or ankle-foot orthosis will alter the corresponding joint impedance while its own weight will also influence the gait. The magnitude and importance of these additional or mixed effects depends on the actual weight and body part to which the weight is added. These interferences between interventions should be considered when designing an experiment, as they might have implications for the clinical condition being emulated.

In general, small interventions result in adaptation towards a gait pattern back to kinematic symmetry. They induce alterations on muscle activation patterns, resulting in slight changes in joint torques, reaction forces or joint kinematics. If retrieving a symmetrical gait movement is not possible, then spatiotemporal parameters will change as well. Either spatial parameters like step length, or temporal parameters like step time, or both will deviate from a symmetric pattern. Modification of the participant's anatomy was mostly done by inducing a leg length inequity. This caused asymmetry in both spatial and temporal gait parameters and GRF asymmetry besides asymmetric joint kinematics. Asymmetric loading of the participant's body was mainly done with a unilateral ankle weight, also inducing asymmetry in spatial and temporal gait parameters. Modifications of the participant's joint impedance gave mixed results depending on the used interventions. Passive and active ankle-foot orthoses were the most examined interventions in this category and caused limited GRF asymmetry and asymmetry of temporal, whether combined with spatial, gait parameters, or not. Manipulations of the participant's sensory feedback was mostly materialized with unilateral vibrotactile stimulation. Depending on the location of application, asymmetry could be reported in both spatial and temporal gait parameters. Manipulation of the environment was done with a split-belt treadmill, also causing asymmetry in spatial and temporal gait parameters, though this tends to be mitigated after an adaptation period.

To induce significant asymmetry, it is thus important to select the right parameters for a given perturbation type. The used scoring system tends to promote the use of an artificial leg length inequity as the most favorable intervention to generate asymmetric gait patterns in overall. In this case, our recommendation would be to use insoles of at least a couple of cm's high. Yet depending on the objectives of a given experimental study, other interventions might be more appropriate. Bigger effects are to be expected with added unilateral weights of about 10% of the total body weight. Altering the natural arm swing of the dominant arm would result in a higher chance of asymmetry compared to the non-dominant arm. We would advise against the use of crutch(es) because of low reproducibility of the results and the need for high cooperation of the subjects. Moreover, they were among the few techniques with confirmed weak effects on the ground reaction forces, along with unilateral shoulder loading and the use of an active ankle-foot orthosis. We would also advise against the use of vibrotactile, electrical or auditory stimulation, for now, since the evidence on these techniques and their capacity of inducing asymmetrical gait are mitigated. We would moreover advise against the use of combinations of interventions since the limited research on this topic showed no additional effects, although more evidence is needed to support this last recommendation.

Besides the asymmetry-inducing procedure, reported asymmetries also depend on the equipment used to collect data and the experiment duration. Firstly, differences in experimental set-up, e.g., using a forearm cast or using a bag as a unilateral arm weight, might influence the results considering that there is most likely a difference in mass and inertia between a cast and a bag and both objects might additionally influence arm movement in a different way. Secondly, for every intervention, there is an adaptation phase to what is causing asymmetry in gait pattern. The duration of this learning phase depends on the type of intervention. For example, when adding a unilateral ankle weight, this can take up to 10 min of adaptation to this additional weight before reaching a new steady state (71, 72). In contrast, when walking on a split-belt treadmill, the adaptation period is much shorter (174). This means that the intervention effects are similar neither over time nor over different set-ups and this should be taken into account when designing and performing experiments.

The proposed scoring system enables researchers to select an intervention based on specific criteria. For instance, if the intervention main objective is to alter the pattern of the ground reaction forces without affecting the symmetry of step lengths or phases durations, we would encourage the choice of knee braces or lateral wedge insoles. Similarly, if looking for the intervention with the fastest set-up time and lowest costs, we would advise an intervention with a relative score of 1.00 for applicability, like wearing a unilateral load on one limb or phase-shifted auditory stimuli. However, we recognize that there might be potential biases in our estimates of installation time and cost, that could for instance vary as a function of geographic location and pre-existing laboratory equipment.

### 4.1 Limitations

Asymmetries in healthy walkers were overviewed after application of specific interventions altering the walking pattern. Yet, the gait patterns of real patients were not compared to those of these healthy walkers. Thus, this review cannot outpoint which pathology is best imitated by which intervention. Furthermore, even if an intervention succeeds in modifying the gait profile of healthy subjects to mimic a pathological walking pattern, sensorimotor functions remain fundamentally different between these healthy persons and disabled patients. This will therefore play a role in the evaluation of assistive devices aiming at symmetrizing gait, since the mechanisms behind gait adaptation will be different in both populations.

Besides the interventions included in this review, other interventions might be designed to induce an asymmetric gait pattern in healthy walkers. During the overall search strategy, we found only one article examining the effects of an asymmetric movement support device on muscle activities (189). This study did not investigate or report effects on spatiotemporal parameters, joint kinematics, or kinetics. Therefore, future research needs to be done to investigate if the effects of asymmetric movement support are limited to muscle activity.

Since gait can be described with a large variety of parameters, there is a need for better standardization in the analyses if one wants to compare different types of interventions (190) or different patient populations (25). These interventions should also be compared in future research to real conditions causing asymmetrical gait, enabling the reader to choose an intervention based on the desired medical condition to be examined. As previously stated, experimental conditions should be more standardized, especially with regard to the data collection devices and the duration of the experiments. When designing a study, attention should also be paid to the diversity of confounding factors, like age, physical condition, walking speed and leg dominance of the participants.

Finally, care must be taken regarding the maturity level of the selected intervention. For instance, until now, only a restricted number of studies investigated the constraining of the metatarsophalangeal joints of one foot, although this particular intervention seems promising to significantly influence spatial and temporal symmetry metrics during walking; while, as mentioned above, the absence of additional effects when combining interventions requires more evidence. Another example is the discussion on the minimal LLI inducing kinematic changes in the leg joints. On the other hand, some existing technologies may also indirectly induce gait asymmetry, although this specific effect has not yet been investigated. For example, (191) showed that electrical stimulation below the motor threshold produced illusory movements of the fingers that were sensed by the subject. As the upper limbs proprioception is thus affected, and considering that an induced asymmetrical arm swing reduces lower limbs coordination (113), future research should investigate whether electrical stimulation could also indirectly alter gait, trough proprioception changes.

## 5 Conclusion

In this review, interventions were divided into five parent categories based on the mode of action. Effects did not only differ between categories but also amongst interventions in the same category, implicating that every intervention resulted in a specific set of compensating mechanisms and gait alterations. Yet, as expected, most of the examined interventions caused an asymmetry in joint kinematics and/or muscle activation. Although most interventions alter the gait pattern with their main mode of action, they would also induce mixed effects.

With the proposed scoring system, we aimed at comparing the different interventions in a broad way, enabling the reader to get a quick and rough estimation of pros and cons of each type of intervention. However, not all gait parameters have been examined for the 16 identified interventions and there is a large heterogeneity in methodological approaches in the different articles. To accommodate for this heterogeneity, we proposed a macroscopic “efficacy” score capturing the global effect of a given intervention on the spatiotemporal parameters, joint kinematics, contact forces and joint torques. With the overall score for “applicability”, we aim at comparing the time required to install the perturbing equipment on the participant, and its costs. These two scores therefore provide a rapid reflection of the merits and limitations of each asymmetric gait intervention. Finally, with the overall score for “maturity”, we aim at assessing the quality and amount of the currently available evidence, by scoring the effect regarding the size of the population on which an intervention has been tested, and the number of studies that tested it.

## Footnotes

^1^To date, the impact on muscle activations has not yet been studied for the vast majority of the asymmetrizing methods. Consequently, unless otherwise specified, this particular aspect was not reported in the text.
