# Supplementary material for: Inducing asymmetric gait in healthy walkers: a review
Source: Front Rehabil Sci. 2025 Mar 17;6:1463382. doi: 10.3389/fresc.2025.1463382 (PMC11955677; doi:10.3389/fresc.2025.1463382)
Supplement: Supplementary file 1 [file Datasheet1.pdf]

Inducing asymmetric gait in healthy walkers: a review

Supplementary Material

Gert Van Der Velde[1,2], Henri Laloyaux[2], Renaud Ronsse[2,3*]

[1]Faculty of Medicine, University of Ghent, Ghent, Belgium

[2] Louvain Bionics, Institute of Mechanics, Materials, and Civil Engineering, UCLouvain, Louvain-la-Neuve, Belgium

[3] Institute of Neuroscience, UCLouvain, Brussels, Belgium

Results of the databases search

This supplementary file gathers the methods and results – in terms of number of documents – obtained when searching the publication databases (Pubmed, Embase, Web of Science, Google Scholar, and Scopus) for constructing the publication portfolio of the main review paper. Searches were done according to 16 different perturbation methods, being further sorted into five parent categories, namely: (1) modifications of the participant's anatomy, (2) asymmetric loading of the participant's body, (3) modifications of the participant's joint impedance (4) manipulations of the participant's sensory feedback, and (5) manipulations of the environment.

1 Modifications of the participant's anatomy

The first category consists of interventions that change the anatomy of the subject and hence cause asymmetrical gait. This is done either by inducing a leg length inequity (LLI) or by using lateral wedge insoles.

The five databases were searched for articles about artificial LLI with the search terms that are reported in Table 1. Titles and abstracts were reviewed for the relevance of the studies performed and after the removal of duplicates, 40 articles were kept, and full manuscript was found for 33 of them. Further selection was done based on full text review. Nine articles were excluded because measurements were done on disabled patients. Six articles were excluded since they aimed at examining the effect of LLI on other metrics than those quantifying gait asymmetry, such as oxygen consumption, and were thus irrelevant for this review. This final selection resulted in 18 included articles that examined the effect of an artificially induced LLI in gait parameters and joints biomechanics of healthy people. Another paper was added after examination of the citing papers of this short-list, thus resulting in a total of 19 manuscripts kept for the analyses.

Table 1: Details of the literature search for artificial leg length inequity

Database	Search mode	Combination of keywords	Found publications	Selected based on abstract and title
Pubmed	Advanced	(((healthy) OR (able-bodied)) AND ((leg length inequality) OR (limb length discrepancy))) AND (gait)	41	13

Embase	Quick	(‘limb length discrepancy’ OR ‘leg length inequality’) AND ‘gait’ AND ‘normal human’	28	12
Web of Science	Advanced	((ALL=(leg length inequality)) OR ALL=(limb length discrepancy)) AND ALL=(gait) AND ((ALL=(healthy)) OR ALL=(able-bodied))	17	8
Google scholar	Advanced	gait AND (able-bodied OR healthy) AND ("leg length inequality" OR "limb length discrepancy")	2.5K*	15
* the first 200 publications, sorted by relevance, were reviewed				
Scopus	Basic	"leg length inequality" AND gait AND (healthy OR able-bodied)	301	26
**Grand total**				**74**
**After removal of duplicates**				**40**
Final selection: based on full text and citing papers				***19***

Search terms used to find articles on the effect of lateral wedge insoles can be found in Table 2. Titles and abstracts were reviewed for the relevance of the studies performed and after removal of duplicates, 14 articles were selected, and full manuscript was found for 13 of them. Further selection was done based on full text reviewing. Ten articles were excluded because participants performed the experiments with lateral wedge insoles on both feet, thus with no specific objective to induce gait asymmetry. One article was excluded because the authors examined the effects of a specific type of insole compared to other types of insoles without comparing the results to those of healthy walking and another article was excluded since it examined foot placement and was irrelevant for this review. This finally resulted in 1 included article examining the effect of unilateral lateral wedge insoles on gait and biomechanics of the lower limbs, and no citing paper was considered as being relevant for this study.

Table 2: Details of the literature search for lateral wedge insoles

**Database**	**Search mode**	**Combination of keywords**	**Found publications**	**Selected based on abstract and title**
Pubmed	Advanced	((lateral wedge insoles) AND (gait)) AND ((healthy) OR (able-bodied))	30	8
Embase	Quick	‘lateral wegde insoles’ AND gait	41	2
Web of Science	Advanced	(((ALL=(healthy)) OR ALL=(able-bodied)) AND ALL=(gait)) AND ALL=(lateral wedge insoles)	47	7
Google scholar	Advanced	gait AND (healthy OR able-bodied) AND "lateral wedge insoles"	456	11
* the first 200 publications, sorted by relevance, were reviewed				
Scopus	Basic	(healthy OR able-bodied) AND gait AND "lateral wedge insoles"	14	7
**Grand total**				**35**
**After removal of duplicates**				**14**
Final selection: based on full text and citing papers				***1***

2 Asymmetric loading of the participant's body

The second category groups the interventions that add weight on segment of one body side, to create a shift of the center of mass (COM) of the whole body, as well as possibly of the COM of a limb. In most of the cases, this asymmetrical hacking of the body morphology is done by adding a unilateral ankle weight, a unilateral arm weight, a unilateral shoulder weight, or added weight to the pelvis. In some cases, this weight is remote from the body and applied only during specific gait phases through a cable transmission. Other interventions of this category cause an asymmetrical distribution of the bodyweight without adding extra weight, for instance by using a crutch. Separate searches were conducted for unilateral ankle weight, a more general search of 'asymmetric loading', for unilateral arm weight and unilateral shoulder load, for added weight to the pelvis and for elbow or axillary crutches.

The search terms used to find articles reporting the effect of adding a unilateral ankle weight can be found in Table 3. Titles and abstracts were reviewed for the relevance of the studies and after removal of duplicates, 18 articles were selected, and full manuscript was found for 17 of them. Two articles were excluded since the aim of these studies was to evaluate the accuracy of wearable devices and the effect on gait was not reported. One article examined the effect of bilateral ankle weights and one the effect of a weighted vest. Both were excluded, such as the article that examined the effect of unilateral weight unloading on stepping threshold. This selection resulted in 12 included articles. Another paper was added after examination of the citing papers of this short-list, thus resulting in a total of 13 manuscripts examining the effect of unilateral ankle weight on gait or biomechanics of the lower limbs.

Table 3: Details of the literature search for unilateral ankle weight

Database	Search mode	Combination of keywords	found publications	Selected based on abstract and title
Pubmed	Advanced	((((ankle weight) OR (distal mass)) OR (inertia ankle)) AND (gait)) AND ((healthy) OR (able-bodied))	385	6
	Quick	('ankle weight' OR 'ankle weights' OR 'ankle weighting') AND 'gait'	28	7
	Quick	('distal mass' OR 'ankle mass') AND 'gait'	6	3
Embase	Quick	('inertia' OR 'inertium') AND 'ankle' AND 'gait'	51	2
Web of Science	Advanced	((((ALL=(ankle weight)) OR ALL=(ankle weights)) OR ALL=(ankle weighting)) AND ALL=(gait)) AND ((ALL=(healthy)) OR ALL=(able-bodied))	338	7
Google scholar	Basic	gait asymmetry ankle weight	26K*	10
* the first 200 publications, sorted by relevance, were reviewed				
Scopus	Basic	"ankle weight" AND gait	119	8
**Grand total**				**43**
**After removal of duplicates**				**18**
Final selection: based on full text and citing papers				***13***

Preliminary search revealed that there are alternative techniques to simulate asymmetric gait by asymmetric loading, such as with an asymmetric shoulder loading or a unilateral arm loading. Unilateral loads attached to the hand like a bag or trolley, the forearm, the elbow or the upper arm,

were all merged into the same category called ‘unilateral arm loading’. So, to capture as much as possible of this body of research, another literature search was done with “asymmetric load” as main search term, combined with more specific ones, as reported in Table 4. Titles and abstracts were reviewed for the relevance of the studies performed and after removal of duplicates, 29 articles were selected and full manuscript was found for 29 of them. Several of them were rejected because they did not investigate gait asymmetry: two articles only reported the effect of the intervention on muscle activity, three articles examined the effects on gait wile stepping down a curb or uneven surface, one article examined the effect of the intervention in static conditions, one article examined the effect of intervention on kinematics of the trunk, one article examined symmetrical loading, and one article examined the effects of combining different asymmetrical loads. This selection resulted in 19 included articles examining the effect of an asymmetric load condition on gait or biomechanics of the lower limbs, and no citing paper was considered as being relevant for this study.

Table 4: Details of the literature search for asymmetric load

**Database**	**Search mode**	**Combination of keywords**	**Found publications**	**Selected based on abstract and title**
Pubmed	Advanced	((asymmetric load) OR (unilateral load) OR (hand hold device)) AND (gait)	2019	15
Embase	Quick	('asymmetric load' OR 'unilateral load' OR 'hand hold device') AND (gait)	20	8
Web of Science	Advanced	(((ALL=(asymmetric load)) OR ALL=(unilateral load)) OR ALL=(hand hold device)) AND ALL=(gait) AND ((ALL=(healthy)) OR ALL=(able-bodied))	163	5
Google scholar	Advanced	gait AND (able-bodied OR healthy) AND ("unilateral load" OR "asymmetric load" OR "hand held device")	843*	17
* the first 200 publications, sorted by relevance, were reviewed				
Scopus	Basic	"asymmetric load" AND gait AND (healthy OR able-bodied)	86	13
**Grand total**				**58**
**After removal of duplicates**				**29**
Final selection: based on full text and citing papers				***19***

The search terms used to find articles on the effect of adding a weight to the pelvis are reported in Table 5. Since one of the initial 24 articles identified in the overall search strategy examined the effect of a weight to the pelvis using a cable-driven active tethered pelvic assist device (A-TPAD), this was added as search term as well. Titles and abstracts were reviewed for the relevance of the studies and after removal of duplicates, 11 articles were selected. Eight of them were excluded since the added weight lied in the body plane of symmetry (in anteroposterior direction) and therefore did not induce any asymmetry. One article was excluded since the effect of adding weight was examined in static conditions and thus irrelevant for this review. This selection resulted in two included articles examining the effect of a unilateral added weight to the pelvis on gait or biomechanics. Another paper was added after examination of the citing papers of this short-list, thus resulting in a total of three manuscripts kept for the analyses.

Table 5: Details of the literature search for added weight to the pelvis

Database	Search mode	Combination of keywords	Found publications	Selected based on abstract and title
Pubmed	Advanced	((((A-TPAD) OR (weight pelvis)) OR (inertia pelvis)) AND (gait)) AND ((healthy) OR (able-bodied))	98	5
Embase	Quick	(‘A-TPAD’ OR ‘pelvis weight’ OR ‘inertia pelvis’) AND (gait)	5	1
Web of Science	Advanced	(((ALL=(A-TPAD)) OR ALL=(weight pelvis)) OR ALL=(inertia pelvis)) AND ALL=(gait)	254	7
Google scholar	Advanced	gait AND (able-bodied OR healthy) AND ("A-TPAD" OR "weight pelvis" OR "inertia pelvis")	77	6
Scopus	Basic	("inertia pelvis" OR "weight pelvis" OR "A-TPAD") AND gait	6	3
**Grand total**				**22**
**After removal of duplicates**				**11**
Final selection: based on full text and citing papers				***3***

The search terms used to find articles reporting the effect of using either one or two crutch(es) are reported in Table 6. Titles and abstracts were reviewed for the relevance of the studies and after removal of duplicates, ten articles were selected. One article was excluded for only examining the effect on muscle activation and two articles were excluded because their main objective was to assess the accuracy of specific equipment for gait analysis. One article only examined the effects of crutch assisted walking on posture and one article examined food placement errors without assessing (a)symmetry. These articles were also excluded. This selection resulted in five included articles examining the effect of the use of crutch(es) on gait and biomechanics of the lower limbs, and no citing paper was considered as being relevant for this study.

Table 6: Details of the literature search for crutch(es)

Database	Search mode	Combination of keywords	Found publications	Selected based on abstract and title
Pubmed	Advanced	(((healthy) OR (able-bodied)) AND (gait)) AND (crutch)	52	4
Embase	Quick	Crutch AND normal human AND gait	41	1
Web of Science	Advanced	(((ALL=(healthy)) OR ALL=(able-bodied)) AND ALL=(crutch)) AND ALL=(gait)	74	1
Google scholar	Advanced	gait AND (healthy OR able-bodied) AND crutch	15.7K*	7
* the first 200 publications, sorted by relevance, were reviewed				
Scopus	Basic	(healthy OR able-bodied) AND gait AND crutch	84	5
**Grand total**				**18**
**After removal of duplicates**				**10**
Final selection: based on full text and citing papers				***5***

3 Modifications of the participant's joint impedance

The third category consists of interventions that change the impedance of one or several lower or upper limb joints and hence cause an asymmetrical gait. Typically, this manipulation consists in increasing the joint stiffness and/or damping, potentially up to full joint locking, corresponding in theory to infinite impedance. This can be done by a unilateral knee brace, unilateral metatarsophalangeal joint constraint, an active or passive ankle-foot orthosis (AFO), or by inducing asymmetrical arm swing. In the latter, one arm moves normally, and movement of the other arm is limited so that both arms do not have a similar movement range while walking.

The search terms used to find articles reporting the effect of a unilateral knee brace are reported in Table 7. Titles and abstracts were reviewed for the relevance of the studies and after removal of duplicates, seven articles were selected, and full manuscript was found for six of them. These six articles were included since they have all examined the effect of unilateral knee brace on gait or biomechanics of the lower limbs. Another three papers were added after examination of the citing papers of this short-list, thus resulting in a total of 9 manuscripts kept for the analyses.

Table 7: Details of the literature search for unilateral knee brace

Database	Search mode	Combination of keywords	Found publications	Selected based on abstract and title
Pubmed	Advanced	(((healthy) OR (able-bodied)) AND (gait)) AND (knee brace)	75	6
Embase	Quick	unilateral knee brace effect on gait	13	2
	Quick	unilateral knee extension restriction	37	1
Web of Science	Advanced	((unilateral knee brace) OR (unilateral knee extension restriction)) AND (effect on gait)	17	4
Google scholar	Advanced	effect OR on OR gait "unilateral knee brace"	12	2
	Advanced	effect OR on OR gait "unilateral knee extension restriction"	7	1
	Basic	unilateral knee brace or unilateral knee extension restriction gait	17.2K*	4
* the first 200 publications, sorted by relevance, were reviewed				
Scopus	Basic	effect of unilateral knee brace on gait	14	3
	Basic	effect of unilateral knee extension restriction	1	1
**Grand total**				**24**
**After removal of duplicates**				**7**
Final selection: based on full text and citing papers				***9***

The search terms used to find articles reporting the effect of asymmetrical arm swing can be found in Table 8. Titles and abstracts were reviewed for the relevance of the studies and after removal of duplicates, 16 articles were selected, and full manuscript was found for 15 of them. Several articles were withdrawn after careful inspection: two since the effect of asymmetrical arm swing was examined on another topic than gait, one because it tested different devices to assess arm swing symmetry, one examined arm swing asymmetry in a specific walking condition that is irrelevant to this review because too far away from standard walking, and four because they examined the mechanisms behind arm

swing in baseline walking, thus with no specific objective to induce gait asymmetry. This selection resulted in seven included articles examining the effect of asymmetrical arm swing on gait or biomechanics of the lower limbs or examining normal variability of arm swing. Another paper was added after examination of the citing papers of this short-list, thus resulting in a total of 8 manuscripts kept for the analyses.

Table 8: Details of the literature search for arm swing asymmetry

Database	Search mode	Combination of keywords	Found publications	Selected based on abstract and title
Pubmed	Advanced	((arm swing asymmetry) OR (arm weight)) AND (gait) AND ((healthy) OR (able-bodied))	59	10
Embase	Quick	(‘arm swing asymmetry’ OR ‘arm weight) AND gait	30	6
Web of Science	Advanced	(((ALL=(arm weight)) OR ALL=(arm asymmetry)) AND ALL=(gait)) AND ((ALL= (healthy)) OR ALL=(able-bodied))	93	10
Google scholar	Advanced	gait AND (able-bodied OR healthy) AND ("arm swing asymmetry" OR "weight arm")	300	13
Scopus	Basic	("arm swing asymmetry" OR "arm weight") AND gait	26	8
**Grand total**				**47**
**After removal of duplicates**				**16**
Final selection: based on full text and citing papers				***8***

The search terms used to find articles describing the effect of an active or passive ankle-foot orthosis are reported in Table 9. Titles and abstracts were reviewed for the relevance of the studies and after removal of duplicates, 26 articles were selected, and full manuscript was found for 23 of them. Two articles only examined the effect of the device on muscle activity and three articles examined the different control mechanisms on a computer simulation of an active AFO. One article examined the effect of a newer design of a specific AFO type, one article examined the effect of an AFO in pathological gait, and two articles examined other effects than the ones due to the AFO itself. These articles were all excluded. This selection resulted in 14 included articles examining the effect of an active or passive AFO on gait or biomechanics of the lower limbs, and no supplementary document was considered as being relevant for the present study after examination of the citing papers.

Table 9: Details of the literature search for active and passive ankle-foot orthosis

Database	Search mode	Combination of keywords	Found publications	Selected based on abstract and title
Pubmed	Advanced	(ankle-foot orthosis) AND (gait) AND ((healthy) OR (able-bodied))	115	17
Embase	Quick	‘ankle-foot orthosis’ AND gait AND normal human	72	10
Web of Science	Advanced	(ALL=(ankle-foot-orthosis)) AND ALL=(gait) AND (ALL=(healthy) OR ALL=(able-bodied))	143	11
Google scholar	Advanced	gait AND (healthy OR able-bodied) AND "ankle-foot orthosis"	6.8K*	15
* the first 200 publications, sorted by relevance, were reviewed				

Scopus	Basic	(healthy OR able-bodied) AND gait AND “ankle-foot orthosis”	146	10
**Grand total**				**63**
**After removal of duplicates**				**26**
Final selection: based on full text and citing papers				***14***

Moreover, analyzing the literature related to active or passive ankle-foot orthosis used to induce gait asymmetry revealed that some research has been similarly conducted with active hip devices. Another category was thus added to cover this type of perturbation. We found two papers that reported the use of such devices to induce gait asymmetry in healthy walkers, another one studying the use of a hip-thigh compressive passive sleeve, and a last one reporting the use of an elastic rope attached to the thigh. These papers were grouped together in a manipulation category called “Hip orthosis and impedance modulation”.

Search terms used to find articles describing the effect of unilateral metatarsophalangeal (MTP) joint restriction are reported in Table 10. Titles and abstracts were reviewed for the relevance of the studies performed and after removal of duplicates, 14 articles were selected, and full manuscript was found for 13 of them. Three articles examined the effect of shoe sole stiffness on the MTP joints of the foot and were excluded since they did not report any gait-related outcomes. One article examined the effect of sole stiffness on energy expenditure, one article examined the effect of different boots on gait and another article the effect of sole stiffness on jumping performance. These articles were excluded since none of them intended to induce gait asymmetry. Similarly, three articles that examined MTP joint angles in normal overground walking, the effect of another foot joint restriction, and the effect of a larger boot were further excluded. This selection resulted in four included articles that examined the effect of unilateral MTP joint restriction on gait or biomechanics of the lower limbs, and no supplementary document was considered as being relevant for the present study after examination of the citing papers.

Table 10: Details of the literature search for unilateral metatarsophalangeal joint constraint

**Database**	**Search mode**	**Combination of keywords**	**Found publications**	**Selected based on abstract and title**
Pubmed	Advanced	(metatarsophalangeal) AND ((stiffness) OR (constrain)) AND (gait)	31	2
Embase	Quick	(‘stiffness’ OR ‘constrain’) AND gait AND metatarsophalangeal	52	1
Web of Science	Advanced	(((ALL=(stiffness)) OR ALL=(constrain)) AND ALL=(gait)) AND ALL=(metatarsophalangeal)	38	2
Google scholar	Advanced	gait AND (stiffness OR constrain) AND metatarsophalangeal	15.4K*	7
	/	Related articles (Zhang 2014)	/	6
* the first 200 publications, sorted by relevance, were reviewed				
Scopus	Basic	(stiffness OR constrain) AND gait AND metatarsophalangeal	40	1
**Grand total**				**19**

**After removal of duplicates**	**14**
Final selection: based on full text and citing papers	***4***

4 Manipulations of the participant's sensory feedback

The fourth category consists of interventions that alter the sensory feedback. Either in a unilateral way, and thus causing an asymmetric gait pattern, or by stimulation of the head and neck region. In this category, we found experimental interventions using vibrotactile, electrical, auditory or visual stimulations. Vibrotactile tendon stimulation is indeed known to interfere with proprioception, generating similar responses in the muscle spindles as real movements.

The search terms used to find articles reporting the effect of vibrotactile stimulation are given in Table 11. Titles and abstracts were reviewed for the relevance of the studies and after removal of duplicates, 14 articles were selected with full text available. Three articles that examined the effects of vibrotactile stimulation in static conditions and two articles that examined the long-term effects of vibration training on gait were excluded. One article examined the effect of vibrotactile stimulation in gait initiation and one article in adaptive locomotion. Five articles examined the validation of an assessment device, the effects on brain excitation, the effects on muscle EMG and the effect of vibration on walking direction. All these articles were excluded since they did not focus on gait asymmetry. This selection resulted in six included articles examining the effect of vibrotactile stimulation – either of head and neck, or unilaterally – on gait or on biomechanics of the lower limbs. Another two documents were added after examination of the citing papers of this short-list, thus resulting in a total of 8 manuscripts kept for the analyses.

Table 11: Details of the literature search for vibrotactile stimulation

**Database**	**Search mode**	**Combination of keywords**	**Found publications**	**Selected based on abstract and title**
Pubmed	Advanced	((vibration) AND (gait)) AND ((healthy) OR (able-bodied))	95	8
Embase	Quick	Vibration AND gait AND 'normal human'	171	10
Web of Science	Advanced	(((ALL=(healthy)) OR ALL=(able-bodied)) AND ALL=(gait)) AND ALL=(vibration)	136	6
Google scholar	Advanced	vibration AND gait AND (healthy OR able-bodied)	33.1K*	8
* the first 200 publications, sorted by relevance, were reviewed				
Scopus	Basic	(able-bodied OR healthy) AND gait AND vibration	87	5
**Grand total**				**37**
**After removal of duplicates**				**13**
Final selection: based on full text and citing papers				***8***

The search terms used to find articles reporting the effect of electrical stimulation are given in Table 12. Titles and abstracts were reviewed for the relevance of the studies and after removal of duplicates, 11 articles were selected with full text available. Two articles that simulated gait movement with electrical stimulation instead of adding electrical stimulation while walking were excluded, as well as an article only focusing on producing illusory movements of the hand. Another article was excluded since it only focused on the techniques of electrical stimulation and four articles were excluded since

they only mentioned muscle activation/force alterations because of electrical stimulation. This selection resulted in four included articles examining the effect of unilateral electrical stimulation on general gait parameters, joint kinematics, ground reaction force, joint contact forces or joint torques. Another document was added after examination of the citing papers of this short-list, thus resulting in a total of 5 manuscripts kept for the analyses.

Table 12: Details of the literature search for electrical stimulation

Database	Search mode	Combination of keywords	Found publications	Selected based on abstract and title
Pubmed	Advanced	((electrical stimulation) AND (gait)) AND ((healthy) OR (able-bodied))	220	5
Embase	Quick	‘electrical stimulation’ AND gait AND ‘normal human’	127	4
Web of Science	Advanced	(((ALL=(healthy)) OR ALL=(able-bodied)) AND ALL=(gait)) AND ALL=(electrical stimulation)	309	6
Google scholar	Advanced	"electrical stimulation" AND gait AND (healthy OR able-bodied)	27.9K*	6
* the first 200 publications, sorted by relevance, were reviewed				
Scopus	Basic	(able-bodied OR healthy) AND gait AND "electrical stimulation"	210	4
**Grand total**				**25**
**After removal of duplicates**				**11**
Final selection: based on full text and citing papers				***4***

The search terms used to find articles reporting the effect of auditory stimulation are given in Table 13. Titles and abstracts were reviewed for the relevance of the studies and after removal of duplicates, 14 articles were selected, and full manuscript was found for 13 of them. Three articles were excluded since they examined the effect of a cognitive task instead of pure auditory stimulation by itself. Articles were included when they examined the effect of auditory stimulation on general gait parameters, joint kinematics, ground reaction force, joint contact forces or joint torques. This selection resulted in nine included articles examining the effect of auditory stimulation on general gait parameters, joint kinematics, ground reaction force, joint contact forces or joint torques. Four more documents were added after examination of the citing papers of this short-list: three reporting also auditory stimulation, and a single one reporting a visual-type perturbation. Both were included within this category – thus renamed “Auditory or visual stimulation” – resulting in a total of 13 documents kept for the analyses.

Table 13: Details of the literature search for auditory stimulation

Database	Search mode	Combination of keywords	Found publications	Selected based on abstract and title
Pubmed	Advanced	((auditory stimulation) AND (gait)) AND ((healthy) OR (able-bodied))	105	6
Embase	Quick	‘auditory stimulation’ AND gait AND ‘normal human’	53	4

Web of Science	Advanced	(((ALL=(healthy)) OR ALL=(able-bodied)) AND ALL=(gait)) AND ALL=(auditory stimulation)	105	7
Google scholar	Advanced	"auditory stimulation" AND gait AND (healthy OR able-bodied)	3.4K*	6
* the first 200 publications, sorted by relevance, were reviewed				
Scopus	Basic	(able-bodied OR healthy) AND gait AND "auditory stimulation"	115	7
**Grand total**				**30**
**After removal of duplicates**				**14**
Final selection: based on full text and citing papers				***13***

5 Manipulations of the environment - Split-belt treadmill

The last category consists of interventions that modify the direct surroundings of the subject and hence cause an asymmetrical gait due to these new environmental constraints. This is done by using a split-belt treadmill – i.e. a treadmill with two belts that can drive each leg at a different speed.

The search terms used to find articles reporting the effect of a split-belt treadmill can be found in Table 14. Titles and abstracts were reviewed for the relevance of the studies and after removal of duplicates, 26 articles were selected, and full manuscript was found for 25 of them. Several of them were rejected since this they did not focus on gait asymmetry: two articles used a split-belt but both belts had the same speed, two articles examined the effect of training on a split-belt treadmill, and two articles focused on the fast adaptation process with belts changing speed every couple of minutes. Another four articles that examined other parameters than gait or lower limb biomechanics were also rejected. This selection resulted in 15 included articles describing the effect of walking on a split-belt treadmill on gait or biomechanics of the lower limb, either in itself or in a situation where the subjects had to do another task simultaneously. After examination of the citing papers of this short-list, we found three supplementary recent references using split-belt treadmill and reporting results about gait asymmetrization, resulting in a total of 18 manuscripts kept for the analyses.

Table 14: Details of the literature search for split-belt treadmill walking

**Database**	**Search mode**	**Combination of keywords**	**Found publications**	**Selected based on abstract and title**
Pubmed	Advanced	(((healthy) OR (able-bodied)) AND (gait)) AND (split-belt treadmill)	107	10
Embase	Quick	'split belt treadmill' AND gait AND 'normal human'	71	12
Web of Science	Advanced	(((ALL=(healthy)) OR ALL=(able-bodied)) AND ALL=(split-belt treadmill)) AND ALL=(gait)	128	12
Google scholar	Advanced	gait AND (healthy OR able-bodied) AND "split- belt treadmill"	1.9K*	10
* the first 200 publications, sorted by relevance, were reviewed				
Scopus	Basic	(healthy OR able-bodied) AND gait AND "split-belt treadmill"	105	10
**Grand total**				**54**
**After removal of duplicates**				**26**
Final selection: based on full text and citing papers				***18***
